# Cigarette Consumption and Cigarette Smoking Prevalence Among Adults in Kansas

**DOI:** 10.5888/pcd12.150011

**Published:** 2015-06-11

**Authors:** John S. Neuberger, Sue Min Lai

**Affiliations:** Author Affiliation: Sue Min Lai, University of Kansas Medical Center, Kansas City, Kansas.

## Abstract

**Introduction:**

Recent tobacco prevention and cessation activities have focused on nonsmoking ordinances and behavioral changes, and in Kansas, the overall prevalence of cigarette smoking among adults has decreased. The objective of this study was to determine whether overall cigarette consumption (mean annual number of cigarettes smoked) in Kansas also decreased.

**Methods:**

Data on cigarette smoking prevalence for 91,465 adult Kansans were obtained from the Behavioral Risk Factor Surveillance System survey for 1999 through 2010. Data on annual cigarette consumption were obtained from the 2002 and 2006 Kansas Adult Tobacco Survey and analyzed by totals, by sex, and by smoking some days or smoking every day. Linear regression was used to evaluate rate changes over time.

**Results:**

Among men, but not women, cigarette smoking prevalence decreased significantly over time. The prevalence of smoking every day decreased significantly among both men and women, whereas the prevalence of smoking on some days increased significantly for women but not men. For current smokers, the mean annual number of cigarettes consumed remained the same.

**Conclusion:**

The decline in overall smoking prevalence coupled with the lack of change in mean annual cigarette consumption may have resulted in a more intense exposure to cigarettes for the smoking population. The significant increase in some day use among women indicates a need for additional prevention and education activities; the impact on future lung cancer incidence rates needs further investigation.

## Introduction

Overall adult smoking rates are decreasing in the United States for both men and women. This phenomenon has also been observed in Kansas. It is important to know whether recent tobacco prevention and cessation activities, which have focused on nonsmoking ordinances and behavioral changes, have resulted in reductions in cigarette consumption.

Recent prevention and cessation activities in Kansas include tobacco counseling for the general population, smoking cessation activities in private organizations and hospital settings, general community education, support groups for patients hospitalized at numerous area hospitals, an American Lung Association *Freedom from Smoking* course, an Internet-based smoking cessation course, focused outreach to American Indian and Alaska Native populations that are state residents, local clean indoor air ordinances for workplaces and public places, media campaigns on the hazards of exposure to secondhand smoke, advocacy activities of the Tobacco Free Kansas Coalition, and state and federal excise taxes on cigarettes. Individuals can take various courses online, in person at a clinic, or through self-help workbooks. Additional help is also available for teenagers and children. The state’s Chronic Disease Risk Reduction Program has assisted in such areas as youth and adult smoking, implementation of local clean air ordinances, and promotion of the Kansas Tobacco Quitline. These activities have been in place for more than 10 years. However, the effectiveness of these Kansas-specific measures is not described in the scientific literature.

Kansas’ excise tax on cigarettes increased from $0.24 to $0.79 per pack from 1995 to 2009. The $0.79 rate has not changed since 2003; the state’s excise tax rate ranks 36th in the nation (1). The federal excise tax on cigarettes increased from $0.24 to $1.01 per pack from 1997 to 2007. Tobacco prevention spending by the state of Kansas was only 7.8% of the minimum amount recommended by the Centers for Disease Control and Prevention in 2007 (2).

Before 2010, Kansas had 36 city and 3 county clean indoor air ordinances, with the first comprehensive municipal ordinance beginning in 2004. The various ordinances were a hodgepodge of requirements, loopholes, and exemptions. These exemptions included allowing smoking in restaurants after 9 pm; allowing certain bars and restaurants to buy out of the regulations; a lack of distance requirements for smoking around doors, operable windows, and air handling systems; allowing smoking in hotels and motels; and exempting certain private clubs (3). A comprehensive state statute, the Kansas Clean Indoor Air Act, went into effect on July 1, 2010, six months before the final data collection period for smoking prevalence. The state law has numerous exemptions; for example, 20% of the state’s hotel and motel rooms and casino gaming floors are exempted.

From 1997 to 2006, cigarette smoking prevalence among adults (aged ≥18 y) decreased significantly among men in 29 of the 51 US reporting jurisdictions (2). Among women, smoking prevalence declined significantly in 30 jurisdictions and increased significantly in one. In Kansas, the annual cigarette smoking rate decreased significantly by 3.0% among men and nonsignificantly by 1.0% among women. No information is available on cigarette consumption in Kansas for that time.

According to Behavioral Risk Factor Surveillance System (BRFSS) survey data from 1996 to 2012, cigarette smoking prevalence declined significantly among men in 39.8% of 3,127 counties and among women in 16.2% of counties (4). A study using 2005–2010 National Health Interview Survey (NHIS) data reported that the proportion of adult daily smokers who smoked 1 to 9 cigarettes per day increased, whereas the proportion who smoked 30 cigarettes or more per day decreased (5). Neither survey provided data on smoking on some days. Another study using NHIS data reported that smoking prevalence among US adults declined from 20.9% in 2005 to 17.8% in 2013, and the proportion of daily smokers declined from 16.9% to 13.7% (6); data on cigarette consumption were not provided.

The objective of our study was to examine cigarette consumption (ie, mean annual number of cigarettes smoked) and smoking prevalence among adults in Kansas using the most recent data available. Our hypothesis was that cigarette consumption decreased as smoking prevalence decreased. 

## Methods

Data on cigarette smoking prevalence in Kansas were obtained for 1999 through 2010 from the BRFSS file on the Kansas Department of Health and Environment website (7). Two survey questions were used by the BRFSS to collect data on smoking prevalence: “Have you smoked at least 100 cigarettes in your entire life?” and “Do you now smoke cigarettes every day, some days, or not at all?” Sample sizes ranged from approximately 4,000 respondents (2000–2003) to approximately 8,000 (2004–2008). The BRFSS response rate in Kansas for 1999 through 2010 ranged from a low of 47.6% in 2000 to a high of 66.3% in 1999. In 2010 the response rate was 59.2%. Details on the survey were described previously (8). 

Data on cigarette consumption were obtained from the 2002 and 2006 Kansas Adult Tobacco Survey (ATS). The ATS asked the 2 BRFSS smoking-related questions, plus 3 additional questions: “On the average, about how many cigarettes a day do you now smoke?,” “During the past 30 days, on how many days did you smoke cigarettes?,” and “On the average, on days when you smoked during the past 30 days, about how many cigarettes did you smoke a day?” Both BRFSS data and ATS data were used because neither source alone offered both prevalence and consumption data and because no single longitudinal study on prevalence or consumption offered the kind of data needed for our analysis.

We calculated mean annual cigarette consumption for smoking every day (number of cigarettes per day × 365) and for smoking some days (number of days of smoking in the past 30 days × the mean number of cigarettes smoked per day × 12 months). To obtain a representative set of population data, all responses were population weighted by age, sex, and race (8). The ATS survey methodology is unpublished but has been stated to be identical to the BRFSS surveys in 2002 and 2006. The ATS covered the entire state and had the same sample sizes as the BRFSS in both years. In both surveys, potential respondents who were not reached after 15 call attempts were categorized as nonrespondents.

Prevalence rates and 95% confidence intervals (CIs) were weighted by age, sex, and race to population characteristics (8). Population data were obtained from the 2000 US Census. We used linear regression for the trend analysis of the 1999–2010 BRFSS data; *P* values were calculated for sex after adjusting for year. For annual cigarette consumption, differences by sex and by year were tested by using the *t* test adjusted for survey design and weight. Significance was determined as *P* < .05, using a 2-tailed test. We did not adjust for multiple comparisons. All statistical analyses were performed using SAS version 9.3 (SAS Institute Inc). The project was approved by the Kansas Department of Health and Environment in 2011.

## Results

**Figure 1:**
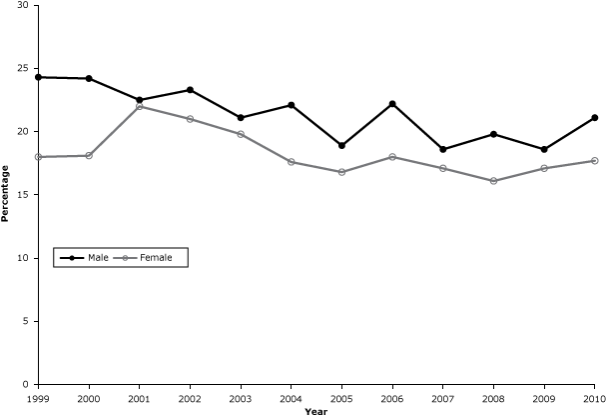
Prevalence of current cigarette smoking among adults in Kansas, 1999–2010. The prevalence decreased significantly among men (*P* = .003) but not among women (*P* = .055). Source of data: Kansas Behavioral Risk Factor Surveillance System. **Table:** 

Data on cigarette smoking prevalence were based on responses from 56,064 women and 35,401 men. Cigarette smoking prevalence was higher among men than among women from 1999 through 2010 (*P* < .001) (Figure 1). The prevalence decreased significantly among men from 24.3% in 1999 to 21.1% in 2010 (*P* = .003), a 12.8% reduction, whereas the prevalence decreased (but not significantly) among women from 18.0% to 17.7% (*P* = .055), a 2.2% reduction 

Year	Men, %	Women, %
1999	24.3	18.0
2000	24.2	18.1
2001	22.5	22.0
2002	23.3	21.0
2003	21.1	19.8
2004	22.1	17.6
2005	18.9	16.8
2006	22.2	18.0
2007	18.6	17.1
2008	19.8	16.1
2009	18.6	17.1
2010	21.1	17.7

**Figure 2:**
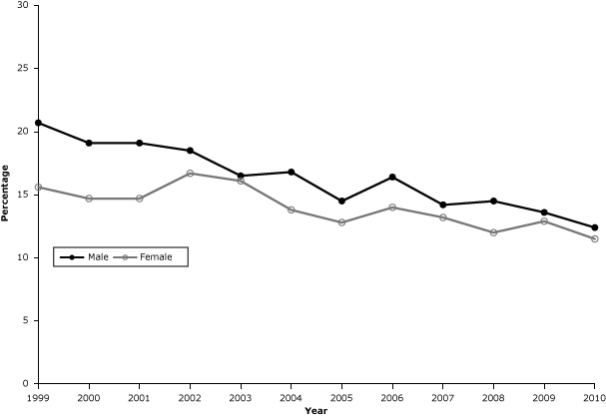
Prevalence of cigarette smoking every day among adults in Kansas, 1999–2010. The decline in smoking rates was significant among men (*P* = .001) and women (*P* < .001). Source of data: Kansas Behavioral Risk Factor Surveillance System. **Table:** 

Among men, the prevalence of every day smoking (Figure 2) decreased from 20.7% in 1999 (95% CI, 18.3%–23.1%) to 12.4% in 2010 (95% CI, 10.8%–14.0%) (*P* = .001). Among women, the rate decreased from 15.6% in 1999 (95% CI, 14.0%–17.2%) to 11.5% in 2010 (95% CI, 10.3%–12.7%) (*P* < .001).

Year	Men, %	Women, %
1999	20.7	15.6
2000	19.1	14.7
2001	19.1	14.7
2002	18.5	16.7
2003	16.5	16.1
2004	16.8	13.8
2005	14.5	12.8
2006	16.4	14.0
2007	14.2	13.2
2008	14.5	12.0
2009	13.6	12.9
2010	12.4	11.5

**Figure 3:**
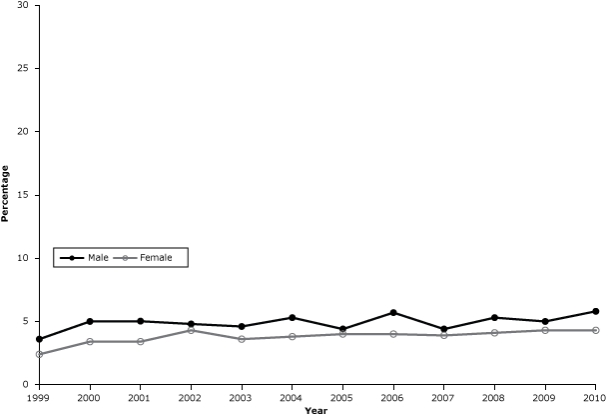
Prevalence of cigarette smoking on some days among adults in Kansas, 1999–2010. The increase was significant among women (*P* = .003) but not among men (*P* = .06). Source of data: Kansas Behavioral Risk Factor Surveillance System. **Table:** 

Among men, the prevalence of smoking on some days fluctuated (Figure 3), ranging from 3.6% in 1999 (95% CI, 2.6%–4.6%) to 5.8% in 2010 (95% CI, 4.4%–7.2%) (*P* = .06). Among women, the prevalence of smoking on some days increased from 2.4% (95% CI, 1.7%–3.1%) to 4.3% (95% CI, 3.5%–5.1%) (*P* = .003).

Year	Men, %	Women, %
1999	3.6	2.4
2000	5.0	3.4
2001	5.0	3.4
2002	4.8	4.3
2003	4.6	3.6
2004	5.3	3.8
2005	4.4	4.0
2006	5.7	4.0
2007	4.4	3.9
2008	5.3	4.1
2009	5.0	4.3
2010	5.8	4.3

Data on mean annual cigarette consumption were based on responses from 1,114 respondents (542 men and 572 women) in 2002 and 3,684 respondents (1,768 men and 1,916 women) in 2006. From 2002 to 2006, the prevalence of smoking every day decreased from 18.5% to 16.1% among men and from 16.7% to 14.0% among women (Figure 2).

The weighted mean annual cigarette consumption per person among all adult smokers in Kansas was 5,150 cigarettes in 2002 and 5,290 cigarettes in 2006; the difference in mean consumption by year was not significant (*P* = .71) (Table). Men consumed more cigarettes than did women in each smoking category. Cigarette consumption overall averaged 14.1 to 14.5 cigarettes per day.

**Table:** Mean Annual Cigarette Consumption Among Smokers in 2002 and 2006, Kansas Adults^a^

Year/Sex	Total Number of Survey Respondents With Complete Information for Calculation	Weighted Mean Annual Consumption per Person (95% CI)
**2002**		
**Men**		
Current	214	5,756 (4,925.6–6,585.4)
Every day	175	7,133 (6,306.4–7,960.4)
Some days	39	977 (484.0–1,469.7)
**Women**		
Current	260	4,480 (3,971.6–4,988.8)
Every day	215	5,425 (4,943.1–5,908.3)
Some days	45	872 (498.8–1,245.1)
**Total Current**	474	5,150^b^ (4,648.7–5,650.8)
**2006**		
**Men**		
Current	579	5,415 (4,861.7–5,968.9)
Every day	464	6,446 (5,799.8–7,092.8)
Some days	115	1,465 (935.5–1,994.4)
**Women**		
Current	686	5,137 (4,627.0–5,647.4)
Every day	570	6,056 (5,526.1–6,586.6)
Some days	116	648 (448.4–848.4)
**Total Current**	1,265	5,290^b^ (4,910.0–5,669.7)

a Data source: 2002 and 2006 Kansas Adult Tobacco Survey.

b No significant difference between weighted means for each year (*P* = .71).

Among men, annual consumption among every day smokers decreased from 7,133 (95% CI, 6,306.4–7,960.4) cigarettes to 6,446 (95% CI, 5,799.8–7,092.8) cigarettes (*P* = .20). Annual consumption among some day smokers increased from 977 (95% CI, 484.0–1,469.7) cigarettes to 1,465 (95% CI, 935.5–1,994.4) cigarettes (*P* = .18).

Among women, annual consumption among every day smokers increased from 5,425 (95% CI, 4,943.1–5,908.3) to 6,056 (95% CI, 5,526.1–6,586.6) cigarettes (*P* = .08). Consumption among some day smokers decreased from 872 (95% CI, 498.8–1,245.1) to 648 (95% CI, 448.4–848.4) cigarettes (*P* = .29).

## Discussion

From 1999 through 2010 current cigarette smoking prevalence among Kansas adults decreased by 12.8% among men and 2.2% among women, compared with a 17.9% decrease nationally for both sexes combined (9). Prevalence data from the Kansas BRFSS and ATS are likely to be similar to each other because the sampling frame and catchment areas are similar for both studies. The rate of decrease in Kansas is less than what might be expected and desirable. The decrease in smoking prevalence among women in our study is in general agreement with an earlier national report on smoking prevalence (2). However, our data provide information on smoking on some days, which was not provided in the national report. The gender gap in the percentage of every day smokers narrowed over time in our study, similar to overall changes in the United States during the past 6 decades (10).

Overall smoking prevalence among men decreased significantly. This decrease included a significant decline in every day smoking and a nonsignificant increase in smoking on some days. Among women, the overall smoking prevalence did not decline significantly. Among women, the trend also included a significant decline in every day smoking and a significant increase in some day smoking, which was unexpected. This increase in some day smoking highlights the need for women to never start smoking.

The total number of cigarettes consumed annually by all adult smokers in Kansas was the same in 2002 and 2006. The decline in smoking prevalence during this period may have resulted in a more intense period of smoking exposure for the overall smoking population.

Given that cigarette consumption in Kansas averaged 14.1 to 14.5 cigarettes per day and assuming that as much as 20 mg of tar is inhaled with each cigarette (11), a substantial amount of tar is inhaled by every day smokers in Kansas, particularly over a 20-year period. For some day smokers, a significant fraction of tar could still be deposited. Although inhaled particles may be partially removed over time, pulmonary particle retention can be as high as 80% to 85% if chronic obstructive pulmonary disease is present (12). Peripheral parts of the lung (eg, alveolar regions) lack ciliated epithelium and mucus-secreting cells (11), possibly resulting in particle retention and increased risk for adenocarcinoma (13).

The effect of possibly having a shorter but more intense period of smoking exposure on future lung cancer rates is unknown. In a recent review and study in Norway, the risk for lung cancer among light smokers was higher in women than men (relative risk among women = 5.03, 95% CI, 1.81–13.98 vs relative risk among men = 2.79, 95% CI, 0.94–8.28) (14,15), and the lung cancer risk among light smokers, although lower than that of regular smokers, was elevated (14,15). The definition of light smoking varies, ranging from 1 to 39 cigarettes per week to less than 1 pack per day. The review could not evaluate rates of intermittent smoking because there is no consistent definition of intermittent smoking in the literature and concluded that the “published cohort studies lack a specific focus on intermittent smoking and tend to underrepresent minority populations, in which this type of tobacco use is most prevalent” (14). Studies of intermittent smokers in Finland and Denmark did not find a significant increase in lung cancer incidence (16,17).

This study has at least 7 limitations: 1) types of smoking other than cigarette smoking are not included, 2) the use of self-reported data, 3) the limited number of years for consumption data, 4) the response rate of the survey, 5) the use of a cross-sectional survey design, 6) sample sizes, and 7) age of the data.

Information on cigar and pipe smoking were unavailable in the BRFSS and ATS, a potential gap in the assessment of tobacco exposure, particularly for men. The data do not indicate whether people are switching from cigarettes to a different type of smoking (ie, cigars, pipes, chewing tobacco, or e-cigarettes) or to dual usage to compensate for switching from smoking every day to smoking on some days.

Prevalence data are based on self-report, suggesting the possibility of recall or social desirability bias. However, a study of self-reported smoking status in Canada, which included measures of urinary cotinine, found a greater than 90% concordance between reported smoking status and urinary cotinine concentrations (18). The authors of the Canadian study caution, however, that 1) the response rate was only 52%, 2) both daily and occasional smokers were classified as smokers, and 3) the measurement of urinary cotinine may be inappropriate for occasional smokers. Similarly, in another study that included data from 3 countries, self-reported cigarette smoking prevalence was compared with either serum cotinine levels or salivary cotinine levels (19). Self-reported smoking prevalence underestimated true tobacco smoking prevalence by 0.6% in the United States, 2.8% in England, and 4.4% in Poland. Information on occasional smokers was not provided. However, using serum cotinine measurements, one of us detected numerous false negative reports of smoking in Kansas during 2006 through 2008 (S.M.L., unpublished data, 2006–2008).

Annual consumption information was based on 2 years of data. Additional cigarette consumption data for 1999–2001, 2003–2005, and 2007–2010 would have been helpful in evaluating trends, but these data were not available.

The BRFSS response rates in Kansas, ranging from 47.6% to 66.3%, are somewhat better than those for the nation as a whole. The median overall BRFSS response rate in 2004 across the 53 states and territories was 41.2% (range, 22.0%–63.4%) (20). Data on smoking prevalence and cigarette consumption represent all Kansans whether or not they answered the survey. Estimates were derived from the sampling design and weights were applied. We had no health or demographic information on people who refused to participate or did not answer the phone. Fifteen call attempts were made before designating a potential respondent as a nonrespondent. Although survey methodology for the ATS is stated to be similar to that for the BRFSS, details on ATS survey methodology are not published.

This was a cross-sectional survey, not a study over time of the same individuals. Thus, trends in smoking cessation might vary by individual age cohort, even if the overall trend is representative of the total population. A single longitudinal study would have been preferred but did not exist. Similarly, a single cross-sectional study with questions on both smoking prevalence and smoking consumption did not exist. Although the cigarette smoking questions were the same over time, the sample sizes varied. For 1999–2001 and 2003–2008, the Kansas BRFSS was conducted using disproportionate stratified sampling methodology, which considers the entire state as a single geographical stratum. In 2002, the sampling method used was only slightly modified from 2001. Beginning in 2009, the sampling method was modified by implementing disproportionate stratified sampling methodology that included selection of landline telephone numbers in 10 geographic strata consisting of county groupings instead of random selection of telephone numbers from the entire state as a single geographic stratum. However, the survey methods used were the same as those used in previous years, and the analysis included data weighting (3).

A relatively small number of people reported smoking on some days in 2002. Information was available from fewer than 40 men and 50 women, and these small numbers may have affected data stability and generalizability. However, the analysis was population weighted.

The data cover 1999 through 2010 for prevalence and 2002 through 2006 for consumption. New data are needed to determine recent changes.

Our study aimed to test the hypothesis that the mean annual number of cigarettes smoked decreased as smoking prevalence decreased. We found that while smoking prevalence declined, cigarette consumption among smokers did not. This could be a problem because exposure and risk are functions of both tobacco consumption and smoking prevalence. The trend in smoking on some days among women increased over time. Although some day smokers may have a reduced risk for lung cancer, their risk may depend on whether they previously accumulated many years of every day smoking or have other environmental or genetic risk factors for lung cancer.

Because of an approximate 20-year delay between smoking prevalence and lung cancer incidence rates (21), we cannot know how a change from smoking every day to smoking on some days will affect future lung cancer rates. No safe level is known for the impact of cigarette smoking on lung cancer, so the extent of sporadic or intermittent smoking on future lung cancer incidence needs further investigation. Follow-up reports of smoking prevalence should routinely include information on annual cigarette consumption for a better estimate of risk.

Analyses like this one can be useful to state policy makers and health departments because they can suggest additional tobacco prevention and control activities. For example, additional education is needed among the smoking population on the need to stop every day smoking. Particular emphasis could be placed on encouraging women never to start. Other activities in Kansas could include increasing excise taxes on tobacco products, strengthening the state’s clean indoor air law, and fully funding the state’s Tobacco Use Prevention Program. Our findings will be shared with the Kansas Department of Health and Environment, and we will work closely with them in developing BRFSS and ATS tobacco-related surveys and tobacco control programs.
